# Evolution of SARS-CoV-2 Key Mutations in Vienna Detected by Large Scale Screening Program

**DOI:** 10.3390/v13102014

**Published:** 2021-10-07

**Authors:** Jakob Thannesberger, Anna Edermayr, Alireza Karimi, Mathias Mueller, Ursula Karnthaler, Richard Gauss, Daniela Penz, Arnulf Ferlitsch, Christoph Steininger

**Affiliations:** 1 Division of Infectious Diseases, Department of Medicine 1, Medical University of Vienna, 1090 Vienna, Austria; christoph.steininger@meduniwien.ac.at; 2 Lifebrain Covid Laboratory GmbH, 1140 Vienna, Austria; anna.edermayr@lifebrain-labor.at (A.E.); alireza.karimi@lifebrain-labor.at (A.K.); mathias.mueller@lifebrain-labor.at (M.M.); 3 City Government of Vienna, State Sanitary Directorate, 1200 Vienna, Austria; ursula.karnthaler@wien.gv.at; 4 City Government of Vienna, Department for Strategic Health Care, 1200 Vienna, Austria; richard.gauss@wien.gv.at; 5 Division of Gastroenterology and Nephrology, Department of Medicine 1, Hospital of St. John of God, 1020 Vienna, Austria; Daniela.Penz@bbwien.at (D.P.); Arnulf.Ferlitsch@bbwien.at (A.F.); 6 Karl Landsteiner Institute for Microbiome Research, 3100 St. Pölten, Austria; 7 Lead Horizon GmbH, 1060 Vienna, Austria

**Keywords:** SARS-CoV-2, COVID-19, Vienna, VOC, B.1.1.7, N501Y, K417N, B.1.351

## Abstract

Currently countries across the globe are preparing for the fourth wave of SARS-CoV-2 infections, which is mainly driven by the rapid spread of novel SARS-CoV-2 variants. Austria and, in particular, the capital city of Vienna, witnessed a disproportionally steep rise in SARS-CoV-2 infection rates during the last wave of infections. By the end of January 2021, the government of Vienna launched an innovative, state-wide SARS-CoV-2 screening program based on PCR analysis of self-collected mouthwash samples. More than 400,000 mouthwash samples were collected in Vienna during the third wave of infection from January to March 2021. All preanalytical and analytical steps were carried out in a highly standardized manner at a single certified testing center. SARS-CoV-2 specific PCR analysis revealed in these samples a positivity rate of 0.43%. The relative proportion of N501Y positive virus samples increased continually to 68% of weekly samples. Mutation K417N was detected only in three samples. With this study, we were able to map the temporal occurrence of SARS-CoV-2 variants in a highly unbiased manner. Positivity rates and variant prevalence rates in this study were lower than in other nationwide programs. The results presented in this study indicate that actual virus prevalence tends to be overestimated by surveillance programs such as results of cluster analysis or contact tracing programs.

## 1. Introduction

In early 2021, the majority of Europe was facing a third wave of SARS-CoV-2 infections. While the overall COVID-19 incidence rate in Austria remained rather stable during January 2021, rates increased rapidly after nationwide lock-down measures were eased by the beginning of February 2021. Especially the eastern parts of the country witnessed a disproportionally steep rise in SARS-CoV-2 infection rates, including the 1.9 million inhabitant city of Vienna [1].

Global public surveillance measures have identified rising numbers of quickly spreading emerging virus variants. The US Center for Disease Control and Prevention has established a catalogue to classify SARS-CoV-2 emerging variants according to their potential changes in transmissibility, disease severity and potential immune evasion [2]. At the time of writing, five variants have been classified as variants of concern (VOC). Among them, variants B.1.1.7 (or alpha), B.1.351 (or beta) and P.1 (or gamma) account for the largest number of infections and have become important drivers of the global SARS-CoV-2 pandemic. Genomic sequencing of VOC has revealed that B.1.1.7, B.1.351 and P.1 all share a single point mutation in the spike gene region that has been linked to increased transmissibility [2]. Due to the extensive monetary, laboratory and personnel capacities required, whole genome sequencing (WGS) is not suitable for screening large sample sizes [3]. PCR-based amplicon sequencing of regions of interest proved to be a more cost efficient and sensitive tool for mutation analysis [4,5]. Hence, analyzing prevalence of SARS-CoV-2 key mutations is a suitable tool for monitoring community spreading of VOC in samples of a large size. 

Likewise, VOC emergence has been linked to rising numbers of SARS-CoV-2 infections in Austria [6]. In the fight against COVID-19, the government of Vienna launched a massive, state-wide screening program based on SARS-CoV-2-PCR testing of self-collected mouthwash samples starting at the end of January 2021. Mouthwash samples are collected by the individuals themselves with the help of a web app which guides test-takers through the process and verifies their identity. The same web app is used to video record the mouthwash procedure to ensure the quality and reproducibility of sample taking. Sampling devices are made accessible free of charge to all people living or working in Vienna. In addition, every SARS-CoV-2-positive sample that showed a Ct-value less or equal to 35 is tested for a VOC. In this study, we present SARS-CoV-2 VOC prevalence data from a mass screening program for COVID-19.

## 2. Materials and Methods

### 2.1. Sample Collection

At the beginning of 2021, health authorities of the city of Vienna initiated a massive COVID-19 PCR-based screening program to fight the resurgence of cases in Eastern Austria. The testing program is based on the promise that a reliable PCR test result is available for everybody living or working in Vienna within a maximum of 24 h after sample collection. People are invited to participate in this program twice a week as regular testing makes sure that persons who have tested positive for COVID-19 can be quarantined and chains of infection can be interrupted early. Test kits for the validated self-collection of mouthwash samples are distributed by local drug stores which makes PCR tests easily available to all participants. Self-collection of samples is performed with support of a dedicated web app, which guides the user through the procedure and determines the identity by verification of an identity card and video surveillance of the procedure (www.lead-horizon.com; accessed on 8 June 2021). The mouthwash is performed for a minimum of 60 s with 2 mL of 0.9% NaCl solution and the sample is then transferred with a paper straw to a transport tube with PBS buffer (Saline viral transport tube, Greiner Bio One, Kremsmünster, Austria). Samples are then packaged in biohazard safe sealed transport bags and cardboard packaging and returned at drug or grocery stores in Vienna, where the samples are collected and transported to the analyzing laboratory.

### 2.2. Sample Processing

Ethics approval for this study was granted by the Ethics committee of the Hospital of St. John of God, Johannes-von-Gott-Platz 1, A-1020, Vienna. All samples are processed within 24 h after collection. Upon sample arrival at the analyzing laboratory, Lifebrain Covid Labor GmbH, the sample tubes are unpacked, scanned for complete data and afterwards the sample material is thermically inactivated at 65 °C for 20 min. For SARS-CoV-2 screening, the samples are pooled within a pool size of 10. Viral RNA is extracted by a fully automated method for magnetic beads-based nucleic acid isolation (chemagic 360, PerkinElmer, Waltham, MA, USA). 

### 2.3. RT-Polymerase Chain Reaction for SARS-CoV-2 Analysis

The detection of SARS-CoV-2 nucleic acid in RNA extracted from the pooled saliva samples is performed with real-time reverse transcriptase polymerase chain reaction (RT-PCR) specific for the SARS-CoV-2 genomic regions ORF1ab and N-gene. Beta-Actin serves as an internal control. RT-PCR is performed using a commercially available, FDA authorized detection kit (Real-Time Fluorescent RT-PCR Kit for Detecting SARS-CoV-2, BGI Genomics Co. Ltd., Shenzhen, China) 

RT-PCR for ORF1ab gene (FAM), N-gene (Cy5) and Beta-Actin (HEX) are carried out using following temperature profile: Hold stage: 20 min on 50 °C; 5 min on 95 °C; 45 cycles of PCR stage: 95 °C for 15 s followed by 20 s on 60 °C and 3 min on 30 °C. 

Only pools that give Ct-values of less or equal 40 for both targets (ORF1ab and N-gene) together with a Ct-value of less or equal 38 for the internal control are counted as positive. Positive pools are resolved, and primary samples are reanalyzed individually by using the qPCR method described above. Positive primary samples with Ct-values of less or equal 35 for either ORF1ab or N-gene are further used for mutation variant screening.

### 2.4. PCR Melting Curve—Mutation Variant Screening

Positive primary samples are screened for the mutation variant N501Y. The 160 bp long fragment is analyzed by RT-PCR melting curve analysis using a commercially available SNIP assay (VirSNiP SARS-CoV-2 Spike N501Y, TIB MolBio, Berlin, Germany). Samples are counted as positive for N501Y when the melting curve analysis shows a melting temperature of 61.2 °C (±2 °C).

All N501Y positive samples are further analyzed for the presence of K417N (88 bp) and V1176F (160 bp) mutations. For this purpose, RNA eluate of N501Y positive saliva samples is further analyzed by commercially available SNIP assays for K417N and V1176F presence (VirSNiP SARS-CoV-2 Spike K417N and VirSNiP SARS-CoV-2 Spike V1176F, TIB MolBio, Berlin, Germany).

Sample is considered positive for K417N or V1176F when the melting curve analysis shows a Tm of 61 °C (±2 °C) or 57.9 °C (±1 °C) respectively. The following thermo cycler profile is used for all 3 melting curve analyses: Hold stage: 55 °C, 10 min; PCR stage: 95 °C for 5 min (1 cycle); PCR stage: 95 °C for 5 s, 60 °C for 30 s and 72 °C for 15 s (45 cycles); final denaturation stage: 95 °C for 30 s, 40 °C for 2 min and 40 °C for 30 s.

## 3. Results

**Figure 1:**
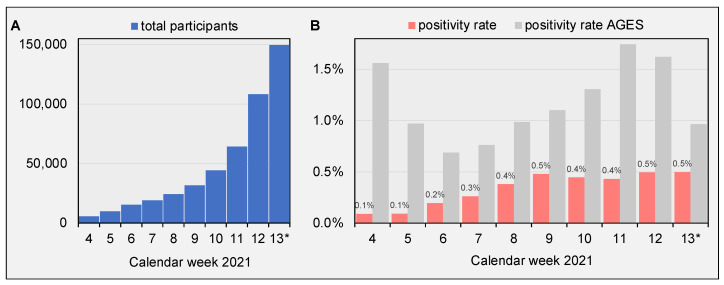
(**A**) Total number of weekly participants. During a study period of 66 days (25 January–31 March), a total number of 400,730 samples were collected. (**B**) Fraction of positive test results of total weekly tests (red bars). Grey bars represent total positivity rate of all tests conducted in Vienna as reported by the Austrian Agency for Health and Food Safety (AGES; https://covid19-dashboard.ages.at; accessed on 8 June 2021). * study period ended at day 3 of calendar week 13 (31 March).

From 25 January to 31 March 2021, a total number of 401,119 samples were collected. The number of samples collected and tested every week increased from 5591 samples at the beginning of the project in calendar week (CW) 4 to 149,740 samples in CW 13 (Figure 1A). Weekly positivity rates increased from 0.1% in CW 4 to 0.5 % in CW 13.

**Figure 2:**
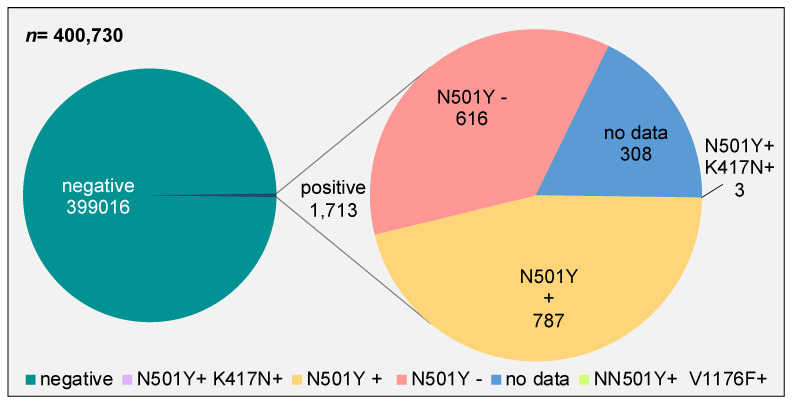
PCR results for VOC key mutations. PCR melting curve analysis was conducted on all SARS CoV-2 positive samples. 0 samples tested positive for mutation V1176F.

In total, SARS-CoV-2 PCR testing was performed on 400,730 samples of which 1714 (0.43%) tested positive (Figure 2). Of all viral strains detected, 787 (45.9%) carried the N501Y mutation (N501Y+), 615 samples (35.9%) tested negative for the N501Y (N501Y-) mutation and the N501Y melting curve analysis remained inconclusive for 312 samples (18.2%). For all N501Y+ samples, we additionally analyzed the presence of the K417N (K417N+) and V1176F (V1176F+) mutations. We identified three samples that were positive for the K417N and the N501Y mutation (N501Y+/K417N+). Mutation V1176F was not detected in any of the positive samples.

**Figure 3:**
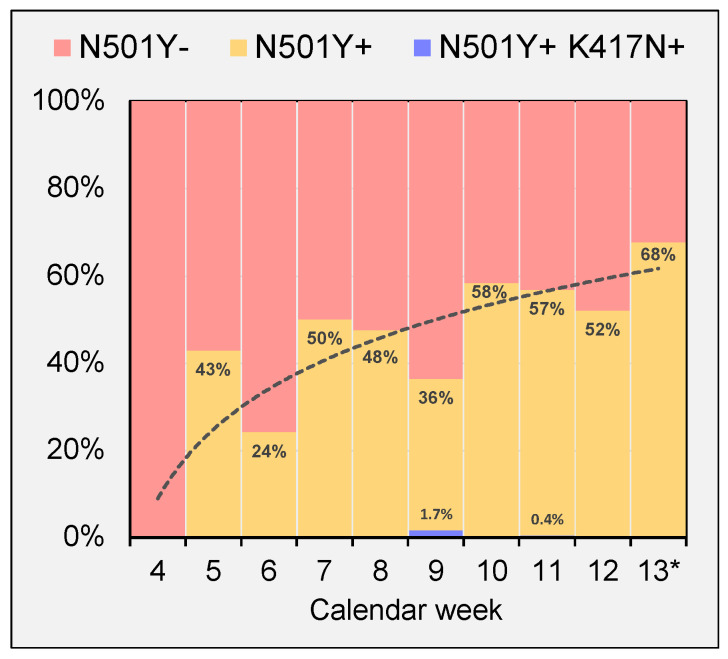
Fraction of key mutations on weekly positive samples. Line represents logarithmic regression for N501Y+ cases (following regression function y = 0.229 ln(x) + 0.0897). * less than 7 days of the indicated week included.

The relative weekly fraction of N501Y+ cases increased over the observed time period from minimum 24% to maximum 68% of samples that gave conclusive results in melting curve analysis (Figure 3). Detection of N501Y+/K417N+ cases were rather occasional events on two separate days, accounting for 1.7% and 0.4% of weekly cases.

## 4. Discussion

In this study, SARS-CoV-2 PCR results of more than 400,000 mouthwash samples from a mass screening program in the city of Vienna were analyzed. All samples have been collected as part of a massive screening program that is based on the validated self-collection of mouthwash samples and high accessibility of PCR test results for the general population of Vienna. The large sample size is a strong indicator for the good compliance of the inhabitants of the city, with an estimated 1.9 million inhabitants. The proposed mass screening program is a well applicable, cost-effective solution for high throughput virus screening.

Large epidemiological SARS-CoV-2 studies often include heterogenous sample entities such as nasal swabs and oropharyngeal swabs collected from populations with highly variable pre-test probability for a positive test [7]. Sample collection is difficult to be standardized as it is usually carried out by a large and diverse group of medical and paramedical staff using variable sampling devices. Comparability of results often is further restricted as analysis of results is usually performed by multiple laboratory centers. For the present study, all preanalytical and analytical steps followed highly standardized protocols. All individuals used identical sampling devices, available free of charge through easily accessible distributors. Sample collection was performed by individuals themselves following a standardized procedure that was video recorded and individually validated by specifically trained personnel. All samples from across Vienna were collected daily and transferred to a single laboratory center for PCR testing to make reliable RT-PCR results available within a maximum of 24 h after sample collection. Thus, observations within this study feature an outstandingly high degree of comparability.

Country-wide testing efforts in Austria are constantly monitored by governmental authorities (i.e., Austrian Agency for Health and Food Safety, AGES) and daily results are made accessible online (https://covid19-dashboard.ages.at; accessed on 8 June 2021). During the observation period of this study, the officially reported positivity rate for Vienna was significantly higher than detected in this screening program, varying between 0.69% and 1.75% of weekly samples (Figure 1B). Officially reported statistics include results of all reported SARS CoV-2 PCR and antigen tests. A large number of tests are conducted specifically for contact tracing, potentially increasing chances for a positive test result. Moreover, current testing policy in Austria primarily utilizes lateral flow antigen tests for testing asymptomatic individuals. PCR testing is mostly performed only as a second step to validate positive antigen tests or in cases where a high accuracy result is required, such as for hospitalized patients or individuals leaving or entering the country. When sample population is biased towards individuals with higher pretest probability for positive test results, SARS-CoV-2 prevalence rates might probably be distorted. We believe that the mean positivity rate of 0.43% observed in this study might give a more representative estimation of SARS-CoV-2 prevalence for the geographically confined area of Vienna, especially with increasing participation rates. The availability of unrestricted, free of charge self-testing possibilities can help authorities to track local infection activity reliably and rapidly. This might provide an objective basis for authorities to evaluate the necessity of socioeconomic countermeasures.

Emerging virus variants have been identified as key factors in COVID-19 dynamics as they influence transmission rates and disease progression. Surveillance efforts were targeted to monitor prevalence of key mutations of designated variants of concern (VOC). Variants carrying the N501Y mutation, such as VOCs B.1.1.7 and B.1.351, were shown to rapidly spread in mainland Europe during early 2021 [2,8]. To monitor emerging VOC, continuous SARS CoV-2 whole genome sequencing programs have been implemented. As virus WGS is a cost expensive procedure, not all samples that were collected by nationwide screening programs can be routinely sequenced. In Austria, WGS is performed routinely only in cases of prolonged COVID-19 duration or occurrence of other clinical complications. Instead, virus key mutations are detected by PCR melting curve analysis. Official VOC prevalence rates in Austria do not differentiate between PCR melting curve analysis and WGS results. Our results show that the fraction of N501Y positive strain infections increased during the observation period. This has also been shown by governmental authorities [1]. Prevalence rates of N501Y mutation reported here (up to 68% of weekly positive cases) are lower than prevalence rates reported by the Austrian Agency for Health and Food Safety (74% of weekly positive cases). This indicates that mutation frequencies are significantly lower in asymptomatic individuals or individuals suffering from mild COVID-19 than in a sample population including individuals suffering from severe COVID-19. This observation has been also confirmed for the K417N mutation where official prevalence rates of up to 1.48% have been reported for Vienna. Within all 400,730 cases analyzed, we detected only three K417N positive strains. Our results indicate that community prevalence of K417N positive SARS-CoV-2 strains such as B1.351 has been low in Vienna during the study period. This is of particular interest as western regions of the country (Tyrol in particular) have experienced B1.351 prevalence rates of up to 21% earlier in 2021 [6,8]. One limitation of this study is that besides N501Y, K417N and V1176F, no other key mutations have been addressed. For future analysis, PCR melting curve analysis can be easily adapted to detect newly identified virus mutations

In conclusion, this study presents results of a SARS-CoV-2 mass screening program carried out during the third wave of infection in Vienna, Austria. The self-collection procedure of mouthwash for PCR analysis has found large and growing acceptance in the general population as more than 400,000 samples within 9 weeks were collected. SARS CoV-2 specific PCR analysis gave a positivity rate of 0.43% and revealed increasing prevalence of N501Y positive virus strains. What differentiates this study from other large-scale SARS-CoV-2 prevalence studies is that (i) all preanalytical and analytic steps have been carried out in a highly standardized manner and that (ii) the free of charge, self-testing design allows us to objectively map SARS-CoV-2 community prevalence.
